# Current and Emerging Reconstituted HDL-apoA-I and HDL-apoE Approaches to Treat Atherosclerosis

**DOI:** 10.3390/jpm8040034

**Published:** 2018-10-03

**Authors:** Eftaxia-Konstantina Valanti, Katerina Dalakoura-Karagkouni, Despina Sanoudou

**Affiliations:** 1 Clinical Genomics and Pharmacogenomics Unit, 4th Department of Internal Medicine, “Attikon” Hospital, Medical School, National and Kapodistrian University of Athens, 11527 Athens, Greece; evalanti@bioacademy.gr; 2 Molecular Biology Division, Biomedical Research Foundation of the Academy of Athens, 11527 Athens, Greece; 3 Department of Biochemistry, University of Crete, Medical School, 71003 Heraklion, Greece; medp2011666@med.uoc.gr; 4 Center for New Biotechnologies and Precision Medicine, Medical School, National and Kapodistrian University of Athens, 11527 Athens, Greece

**Keywords:** atherosclerosis, lipoprotein metabolism, reconstituted HDL, apolipoprotein A-I, apolipoprotein E

## Abstract

Atherosclerosis affects millions of people worldwide. However, the wide variety of limitations in the current therapeutic options leaves much to be desired in future lipid-lowering therapies. For example, although statins, which are the first-line treatment for coronary heart disease (CHD), reduce the risk of cardiovascular events in a large percentage of patients, they lead to optimal levels of low density lipoprotein-cholesterol (LDL-C) in only about one-third of patients. A new promising research direction against atherosclerosis aims to improve lipoprotein metabolism. Novel therapeutic approaches are being developed to increase the levels of functional high density lipoprotein (HDL) particles. This review aims to highlight the atheroprotective potential of the in vitro synthesized reconstituted HDL particles containing apolipoprotein E (apoE) as their sole apolipoprotein component (rHDL-apoE). For this purpose, we provide: (1) a summary of the atheroprotective properties of native plasma HDL and its apolipoprotein components, apolipoprotein A-I (apoA-I) and apoE; (2) an overview of the anti-atherogenic functions of rHDL-apoA-I and apoA-I-containing HDL, i.e., natural HDL isolated from transgenic Apoa1^−/−^ × Apoe^−/−^ mice overexpressing human apoA-I (HDL-apoA-I); and (3) the latest developments and therapeutic potential of HDL-apoE and rHDL-apoE. Novel rHDL formulations containing apoE could possibly present enhanced biological functions, leading to improved therapeutic efficacy against atherosclerosis.

## 1. Introduction

Cardiovascular disease (CVD) is the leading cause of morbidity and mortality worldwide [1]. Coronary heart disease (CHD) is the most common type of CVD and is caused by atherosclerotic occlusion of the coronary arteries [2]. Coronary heart disease causes approximately 7.2 million deaths worldwide, while the annual cost of CHD in the USA alone has been estimated to be over $10 billion [1,2]. Current projections estimate that the prevalence of CHD along with the associated cost of treatment will increase by almost 18% and 43%, respectively, by the year 2030 [2]. 

Atherosclerosis develops over the course of decades as a result of endothelial dysfunction, vascular inflammation, lipid and fibrous component accumulation along with gradual thickening of the blood vessel walls [3,4,5]. Extensive research over the years has led to the development of several low density lipoprotein cholesterol (LDL-C)-lowering therapeutic approaches, including statins, inhibitors of cholesteryl ester transfer protein (CETP), inhibitors of intestinal cholesterol absorption (ezetimibe) and inhibitors of proprotein convertase subtilisin/kexin type 9 (PCSK9) [6,7,8]. However, despite their significant benefits in the clinical setting, each treatment has considerable limitations, such as considerable patient-to-patient variability in lowering LDL-C, which leaves a significant number of patients with suboptimal treatment options [9,10,11,12,13,14,15]. For example, statins, which are the first line LDL-C lowering treatment in CHD patients, lead to optimal LDL-C levels in only 37% of patients [16], while the residual cardiovascular risk despite considerable LDL-C reduction by statin therapy is estimated at 12–22% [17,18,19,20].

New, potentially better targeted approaches are being pursued, aiming to prevent the development and progression of atherosclerosis by targeting atherogenic mechanisms at the level of the arterial wall, which ranges from lipid accumulation and endothelial dysfunction to inflammation and plaque stabilization [21,22,23,24]. One promising research avenue lies in high density lipoprotein (HDL) metabolism and functionality as a means to treat, or ideally prevent, atherosclerosis [7,21,25,26,27,28,29,30]. The failure of several HDL-C raising drugs to decrease the incidence of recurrent cardiovascular risk in CHD patients, despite raising HDL-C [31], has led to the conclusion that HDL functionality is more important that HDL-C levels per se in atheroprotection [7,25,29,32].

## 2. The Atheroprotective Properties of HDL, apoA-I and rHDL-apoA-I

HDL functionality is dependent on its apolipoprotein and lipid composition. Furthermore, it has multiple atheroprotective features, including reverse cholesterol transfer, anti-inflammatory, anti-oxidative, anti-thrombotic, vasodilatory and re-endothelialization related properties [26,33,34,35]. Numerous studies in animal models of atherosclerosis and human patients have shown that HDL inhibits the development of atherosclerosis and even promotes atherosclerotic plaque regression [33,36]. The anti-atherogenic functions of HDL have been attributed to apolipoprotein A-I (apoA-I) to a certain extent, which is the major apolipoprotein component of HDL that participates in the biogenesis, remodeling and signaling of HDL [33]. Studies in apolipoprotein E (apoE) or low density lipoprotein receptor (LDLR) deficient mice have shown that hepatic overexpression of human apoA-I leads to 70% regression of pre-existing aortic atherosclerotic lesions [37]. Consistent with these findings, double deficient mice for apoA-I and LDLR which were fed an atherogenic diet developed more severe atherosclerosis compared to the apoA-I deficient animals [38]. The anti-atherosclerotic effects of apoA-I have been associated with its ability to promote cholesterol efflux from macrophage-derived foam cells in vivo via interactions with the ATP binding cassette subfamily A member 1 (ABCA1) and ATP binding cassette subfamily G member 1 (ABCG1) transporters [37,39]; to reduce arterial wall inflammation [40,41,42]; and to preserve endothelium integrity [43,44,45,46]. Due to these atheroprotective properties of HDL and apoA-I, extensive research efforts have been geared towards the development of HDL and apoA-I targeted therapeutic approaches increasing the levels of functional HDL particles [7,25,29,32,47]. Ongoing clinical trials involve apoA-I and HDL mimetic peptides, apoA-I transcriptional upregulators, de-lipidated HDL as well as in vitro synthesized reconstituted forms of discoidal HDL particles containing phospholipids and apoA-I as the sole apolipoprotein (rHDL-apoA-I) [7,25,29,47,48]. This review is focused specifically on the developments of rHDL-based therapeutic approaches.

The infusion of rHDL-apoA-I particles, which are designed to favor interactions with the ABCA1 transporter, constitute a direct approach to elevate plasma levels of apoA-I and discoidal pre-β HDL particles, resulting in an increase in mature spherical HDL particles and subsequently HDL-C levels [25,49,50,51,52,53]. Moreover, rHDL-apoA-I infusion therapies enhance the atheroprotective functions of HDL, such as cellular cholesterol efflux capacity, while minimizing its off-target effects [11,50,51,54]. A number of rHDL-apoA-I formulations containing either native wild type apoA-I purified from human plasma (CSL-111, CSL-112, CER-001) or apoA-I Milano (MDCO-216), which is a genetic variant of apoA-I whose carriers experience reduced CVD risk despite low HDL-C levels [55], complexed with phospholipids have progressed from in vivo studies to clinical trials [11,50,52,53,54,56,57,58]. In pre-clinical studies, these various rHDL-apoA-I agents have been shown to promote the regression of coronary atherosclerotic plaques in vivo by increasing ABCA1-mediated cholesterol efflux from macrophages [59,60]. However, despite the promising in vivo findings as MDCO-216 [52], CSL-111 [56] and CER-001 [53] infusions increased apoA-I plasma levels and enhanced HDL-C cholesterol efflux capacity in healthy volunteers and CHD patients, they failed to achieve a significant regression in coronary atherosclerosis when tested in clinical trials [11,53,56,57]. In contrast, the infusions of CER-001 in patients with familial hypoalphalipoproteinemia and homozygous familial hypercholesterolemia led to atherosclerosis regression, with CER-001 currently being assessed in a phase III clinical trial [61,62]. Further clinical development of MDCO-216 and CSL-111 has been halted due to prohibitive production costs and adverse effects, respectively, combined with their failure to demonstrate a beneficial effect at the clinical level [11,25,53,57]. CSL-112 is a promising rHDL infusion therapy consisting of purified native human apoA-I combined with phosphatidylcholine, which has a reduced lipid/apoA-I ratio compared to CSL-111 [25]. Numerous phase I and II clinical trials in healthy volunteers [54] and patients with stable atherosclerotic CHD [50] or after an acute myocardial infraction [58] have demonstrated that CSL-112 enhances the levels of apoA-I and pre-β HDL, induces a marked increase in cholesterol efflux capacity and is well tolerated with no evidence of liver toxicity. Despite the favorable clinical trial data, the potential of CSL-112 in reducing major adverse cardiovascular events in high-risk acute coronary syndrome patients remains to be determined in the large phase III AEGIS-II study that is expected to be concluded in 2022 [11].

## 3. The Atheroprotective Functions of apoE

Based on the therapeutic potential of rHDL-apoA-I formulations, the development of additional novel rHDL agents with different apolipoprotein/lipid compositions and enhanced atheroprotective activities holds promise for CHD patients [25,51]. Reconstituted HDL particles containing apoE (rHDL-apoE), which is an apolipoprotein that has pleiotropic atheroprotective functions [48,63], are one of the new rHDL agents under investigation.

Apolipoprotein E is a 34-kDa polymorphic glycoprotein largely secreted by the liver although it can also be locally produced by macrophages in atherosclerotic lesions [64]. The most common isoforms of apoE are apoE2, apoE3 and apoE4, which are the products of three different alleles at a single gene locus [63]. The apoE3 (Cys112/Arg158) is considered to be the most frequently encountered form of apoE [63]. The rarest variant, ApoE2 (Cys112/Cys158), is associated with increased levels of triglyceride-rich lipoproteins (TRLs) and Type III hyperlipoproteinaemia [65,66], while apoE4 (Arg112/Arg158) is associated with increased LDL-C levels [65,66,67,68].

Numerous pre-clinical and clinical studies have demonstrated the pleiotropic anti-atherogenic properties of apoE. The majority of these studies evaluated the atheroprotective functions of all three apoE isoforms (apoE2, apoE3 and apoE4), whereas others focused specifically on apoE3, since it appears to have more beneficial effects as discussed below. ApoE is a component of TRLs and promotes the LDLR dependent hepatic clearance of TRL remnants from the circulation [63]. Moreover, apoE stimulates the hepatic clearance of chylomicron remnants through its binding to the LDL receptor related protein (LRP) or the heparan sulfate proteoglycan (HSPG) receptor [69,70]. Dominant mutations have been identified in apoE that affect its interaction with the LDLR and are associated with higher plasma cholesterol levels, leading to hypertriglyceridemia in mice and type III hyperlipoproteinaemia in humans [63,71]. Consistent with these findings, a homozygous familial apoE deficiency in humans has been associated with a pro-atherogenic lipoprotein profile and atherosclerotic disease [72]. Similarly, apoE knockout (Apoe^−/−^) mice are hypercholesterolemic due to impaired hepatic clearance of TRL remnants, which leads to the development of atherosclerosis even when the mice are fed a low fat diet [71,73]. In contrast, the hepatic overexpression of human apoE3 induced atherosclerosis regression in Apoe^−/−^ mice [74].

In addition to its role in TRL clearance, apoE stimulates hepatic very low density lipoprotein (VLDL) and triglyceride secretion and thus participates in the homeostasis of plasma cholesterol and triglyceride levels [75]. Importantly, apoE induces reverse cholesterol transport from peripheral tissues to the liver [63]. Numerous studies have demonstrated that apoE stimulates cholesterol efflux from macrophages and thus prevents foam cell formation and atherogenesis [76,77,78]. ApoE3 has been associated with higher cholesterol efflux capacity from human monocyte-derived macrophages compared to apoE2 and apoE4 [76]. HDL isolated from Apoe^−/−^ mice demonstrated decreased cholesterol efflux capacity from mouse peritoneal macrophages although this was restored to normal by the administration of exogenous apoE [77]. Similarly, the expression of human apoE3 in macrophages increased the cholesterol efflux capacity from the arterial wall of Apoe^−/−^ mice and limited the development of atherosclerosis [78]. Moreover, apoE can provide protection from atherosclerosis by promoting the in vivo biogenesis of functional spherical apoE-containing HDL particles (HDL-apoE) with the participation of ABCA1 and LCAT (lecithin-cholesterol acyltransferase) independently of apoA-I [79,80]. It has been shown that these HDL-apoE particles can be produced from transgenic Apoa1^−/−^ or double deficient Apoe^−/−^ × Apoa1^−/−^ mice overexpressing human apoE, which accounts for at least some of the atheroprotective properties of apoE, as discussed below [79,80]. Finally, apoE activates the enzymes involved in lipoprotein metabolism, such as hepatic lipase (HL), CETP and LCAT. Thus, it enhances phospholipid hydrolysis in HDL, lipid exchange between VLDL and HDL and conversion of discoidal to spherical mature HDL particles [81,82,83].

Interestingly, several biological functions of apoE that are not directly related to lipid metabolism and transport also contribute to its anti-atherogenic role, such as its anti-inflammatory, anti-oxidative, anti-thrombotic and endothelial repair related properties [84,85,86,87,88,89,90]. Macrophage-derived apoE has been shown to suppress the activation of human umbilical vein endothelial cells (HUVECs) by inhibiting the expression of vascular cell adhesion molecule 1 (VCAM-1) and stimulating intracellular nitric oxide (NO) production [84,85]. In accordance with these findings, it has been shown that apoE3 binding to the ApoER2 receptor attenuates monocyte adhesion to bovine aortic endothelial cells (BAECs) [86]. Interestingly, a recent study showed that apoE expression in the monocytes and macrophages of hypomorphic apoE mice deficient in LDLR expression (Apoe^h/h^LDLR^−/−^ mice) attenuated nuclear factor kappa B (NF-κΒ)-dependent inflammation and atherosclerosis by enhancing cellular miR-146a levels [91]. Moreover, apoE exerts anti-inflammatory actions by inhibiting the activation and proliferation of T lymphocytes [87] as well as by suppressing the migration and proliferation of smooth muscle cells induced by oxidized LDL [88]. In addition, apoE can inhibit lipoprotein oxidation and platelet aggregation and may thereby prevent the accumulation of oxidized LDL on the arterial wall as well as thrombus formation on atherosclerotic lesions [89,90]. Finally, the effect of apoE on endothelial cell functions contributes to its anti-atherogenic role [86,92]. It has been shown that the binding of apoE3 to ApoER2 induces BAEC migration and carotid artery re-endothelialization in mice through the stimulation of NO release [86]. Furthermore, apoE3 also protected HUVECs from apoptosis by suppressing the activation of caspases 3 and 7 [92].

At the clinical level, apoE synthetic mimetic peptides have been studied in phase I and phase II trials to investigate if they significantly reduce plasma cholesterol levels, with Ac-hE18A-NH2 having been licensed under the trade name AEM-28 [48,93,94,95,96]. Their effect against atherosclerosis is now under investigation [48].

## 4. Advantages and Therapeutic Potential of apoE, HDL-apoE3 and rHDL-apoE3

Based on the aforementioned findings, apoA-I and apoE appear to provide protection from atherosclerosis largely through similar functions, including the cholesterol efflux capacity, the anti-inflammatory, anti-oxidative and anti-thrombotic functions as well as their re-endothelialization and plaque stabilizing related properties. However, numerous studies have indicated that the ability of apoE to provide protection from atherogenesis appears to be more pronounced than that of apoA-I [96,97,98,99]. Indeed, in contrast to apoE deficiency, the deficiency of apoA-I does not cause atherosclerosis in mice by itself [96,97]. For example, Apoe^−/−^ mice who were fed a high fat, high cholesterol Western-type diet developed atherosclerosis within 8 to 10 weeks [96,100,101]. Atherosclerotic lesions were also observed in Apoe^−/−^ mice consuming a low fat, low cholesterol chow diet as early as 10 to 12 weeks of age [73,96,101]. On the contrary, no atherosclerotic lesions were evident in Apoa1^−/−^ mice when fed normal chow or even atherogenic diets for 20 weeks [97]. Likewise, although mice that were deficient in the HDL receptor SR-BI (scavenger receptor class B type I) (SR-BI^−/−^) and received a Western-type diet for 20 weeks developed atherosclerosis [100,102], the double deficient Apoe^−/−^ × SR-BI^−/−^ mice, which expressed the endogenous apoA-I, developed severe atherosclerotic lesions as early as 4 to 5 weeks of age when fed a standard chow diet [98,99,100]. Notably, these double knockout mice exhibited an early onset of occlusive atherosclerotic CHD, spontaneous myocardial infarctions, severe cardiac dysfunction and death at a young age (8 weeks) [99]. On the contrary, no atherosclerotic lesions were detected in SR-BI^−/−^ mice at four to seven weeks of age when fed a normal chow diet [98]. Consistent with the above findings that suggest a more profound effect of apoE compared to apoA-I in terms of atheroprotection, the Ac-hE18A-NH2 apoE mimetic peptide was shown to be more effective than the 4F ApoA-I mimetic in reducing the formation of atherogenic lesions in Apoe^−/−^ mice [96]. Additionally, the anti-inflammatory properties of Ac-hE18A-NH2 were reported to be superior to those of 4F [97].

The more profound effect of apoE compared to apoA-I on atheroprotection could at least partly be attributed to certain unique properties of apoE [79]. Unlike apoA-I, apoE can promote hepatic clearance of atherogenic TRL remnants from the circulation, influence hepatic VLDL secretion and activate lipoprotein metabolism related enzymes, which has been discussed above [63,75,81,82,83]. Furthermore, HDL-apoE particles are characterized by significant structural and functional differences compared to apoA-I-containing HDL (HDL-apoA-I) particles [79,80] and could therefore contribute to the enhanced atheroprotective effects of apoE. Although HDL-apoE particles have been studied to a limited extent, the preliminary results seem to be promising [79,80,96,97,98,99,103]. For example, in contrast to Apoe^−/−^ mice which develop atherosclerosis at a young age [96], Apoa1^−/−^ mice do not develop atherosclerosis when fed normal or atherogenic diets [97]. The possible explanations for these observations include a compensatory role of apoE through its multiple atheroprotective functions, including the formation of HDL-apoE particles that could provide protection from atherosclerosis [79]. Consistent with this notion, in the absence of apoE, neither the atheroprotective HDL-apoE particles can be formed nor the atherogenic TRL remnants can be cleared [79]. For instance, the HDL particles derived from the SR-BI^−/−^ × Apoe^−/−^ mice, which lacked apoE, failed to protect the endothelium from atherosclerosis [79,99]. Additionally, the impaired TRL remnant clearance dramatically accelerated atherosclerosis in these mice at four to five weeks of age [99].

A recent study shed light on the distinct atheroprotective properties of HDL-apoE compared to HDL-apoA-I [80]. In specific, in double deficient Apoe^−/−^ × Apoa1^−/−^ mice, the expression of either human apoE3 or apoA-I led to the formation of HDL-apoE3 or HDL-apoA-I particles, which differed in size, apolipoprotein and lipid content as well as HDL functionality [80]. Regarding the apolipoprotein content, apoE3 expression led to the exclusive recruitment of apoC-I on HDL, thus promoting the formation of apoC-I-containing HDL [80]. In contrast, the expression of apoA-I resulted in the formation of HDL particles containing primarily apoA-II and apoC-III [80]. Τhe lipid composition was also different in that the synthesized HDL-apoA-I contained elevated levels of phospatidylserine and phosphatidylglycerol, while the levels of phosphatidylinositol and phosphatidic acid were comparable between HDL-apoA-I and HDL-apoE3 [80]. Importantly, the structural differences between those particles resulted in functionally distinct HDL subpopulations [80]. HDL-apoE3 presented a markedly reduced anti-oxidant potential in vitro and decreased ABCG1-mediated cholesterol efflux capacity from macrophages in vivo [80]. However, HDL-apoE3, but not HDL-apoA-I, reduced TNFα release from macrophages, suggesting that the anti-inflammatory effects of HDL-apoE3 are independent of the presence of apoA-I, while those of HDL-apoA-I are dependent on apoE3 expression [80].

The summary of these findings supports a heightened atheroprotective potential for HDL-apoE3 and consequently, a noteworthy potential in the clinical setting. Preliminary evidence towards this direction has showed that rHDL-apoE interacts with SR-BI and promotes cholesterol efflux [103]. However, further pre-clinical and future clinical studies are needed to demonstrate the potential of rHDL-apoE as a novel therapy for atherosclerosis. Our team is now focusing on the molecular, cellular and in vivo effects of rHDL-apoE with promising results to date (unpublished data).

## 5. Conclusions

A number of rHDL formulations have been developed using apoA-I to enhance the atheroprotective functions of HDL, with the ultimate goal to treat or even prevent atherosclerosis. However, with the exception of CSL-112, these agents were unable to demonstrate a clinical benefit in CHD patients. Nowadays, the encouraging results of CSL-112 pre-clinical and clinical studies, along with knowledge on the pleiotropic atheroprotective properties of apoE3 and the emerging advantages of HDL-apoE3 over HDL-apoA-I, support the potential of rHDL-apoE3 in combating atherosclerosis. Novel rHDL formulations containing apoE3, either used alone or in combination with apoA-I, could possibly present enhanced atheroprotective functions. Further studies are now needed to assess the full potential of rHDL-apoE3 in vitro and in vivo.
